# Fluorescence intensity and fluorescence lifetime measurements of various carbon dots as a function of pH

**DOI:** 10.1038/s41598-023-37578-z

**Published:** 2023-06-30

**Authors:** Wiktoria K. Szapoczka, Adam L. Truskewycz, Tore Skodvin, Bodil Holst, Peter J. Thomas

**Affiliations:** 1 Department of Physics and Technology, University of Bergen, 5007 Bergen, Norway; 2 Department of Biomedicine, University of Bergen, 5009 Bergen, Norway; 3 Department of Chemistry, University of Bergen, 5007 Bergen, Norway; 4 NORCE Norwegian Research Centre AS, 5008 Bergen, Norway

**Keywords:** Nanoparticles, Sensors and biosensors, Fluorescent probes, Optical materials

## Abstract

Measurement and monitoring of pH are essential in both the industry and academia. It is therefore important to continue developing novel, low-cost pH sensors that provide increased accuracy over long periods of time. Particularly promising are sensors based on materials that show pH-dependent fluorescence intensity (FI) and lifetime (FL). Carbon dots (CDs) are emerging as promising candidates because of their low cost, ease of manufacturing, low toxicity, and negligible photobleaching. However, little has been done to quantify the FI and FL values of CDs. Here we report the characterisation of the pH-dependent FI and FL of four novel solvothermal synthesised CDs. The fifth CD is used as a reference sample and was synthesised following a published synthesis. The precursors for the CDs include disperse blue 1 dye, phloroglucinol, *m*-phenylenediamine (*m*-PD), *N*, and *N*-dimethylformamide (DMF). The average diameter size of the CDs ranges from 1.5 to 15 nm. An excitation wavelength of 452 nm with a bandwidth of 45 nm was used to quantify the fluorescence in the pH range 5–9. Three CDs show a decreasing trend in FI with pH, while two CDs show an increasing trend. None of the CDs shows strong FL dependence. The FL changes around 0.5 ± 0.2 ns across the tested pH range. We suggest that the differences in the fluorescence trends can be attributed to the precursors chosen for synthesising the CDs.

## Introduction

PH is a critical parameter in numerous areas of research and industry. Small environmental changes in pH can have significant consequences for living organisms, for example, in enclosed aquaculture systems, where the pH is changed by $\mathrm {CO_2}$ and ammonia levels, as respiratory and metabolic bi-products from fish^1,2^. In nature, ocean acidification alters the seawater carbonate chemistry, causing a dis-balanced carbonate ion saturation, crucial for forming calcium carbonate, the basic building block of skeletons and shells of many marine organisms, including corals, shellfish and plankton^3–6^.

The widespread importance of pH measurements causes a need for suitable pH sensing materials. Currently, the classical pH electrode is the most widely used sensor across various industries^7–9^. The inexpensive pH electrode has several critical disadvantages, including vulnerability to drift (typically 0.25 pH or more over six months^7^), requiring regular re-calibrations^7^. Furthermore, pH electrodes require regular addition of electrolytes to offset consumption and do not function well in high salinity environments due to the instability in the junction potentials at the reference electrode^8,10–12^. Therefore, pH electrodes are better suited for spot sampling than long-term monitoring.

Various sensing technologies have been developed to overcome the limitations of the pH electrode, including Ion Sensitive Field Effect Transistors (requires a reference electrode)^13–15^, spectrophotometric-based sensors (requires periodic refilling of agents, is expensive)^16,17^ and optical sensors.

Optical pH sensor technologies are attracting a lot of interest due to their affordability, low power consumption, and long-term stability^8,18–21^. Optical sensors are particularly interesting in biomedicine, where pH is of great importance in many biological processes, as well as in environmental research and the industry, where one has to deal with ecosystems, and live organisms^18,22–25^. These pH sensors are typically made of a sensing film consisting of a pH indicator immobilised in an ion-penetrable medium and an optoelectronic interrogator unit for probing the film. When the pH of the liquid surrounding the film changes, some optical properties of the indicator change and can be quantified using optoelectronic methods. So far, most studies on pH-sensitive fluorescent materials have focused on FI^22^. A disadvantage of FI-based optical pH sensors is that their response can be strongly influenced by factors such as photobleaching from sunlight or the probe light itself, leaching of the sensor’s indicator from the immobilising medium, background noise from luminescence, and variations in probe light sensitivity^18,19^. Due to these problems, recent research has focused on using the FL as a pH indicator instead of the FI. FL is an intrinsic property of a material and thus is not affected by the factors mentioned above^18,20,21,26–32^, making FL a more stable and reliable trait for prolonged pH sensing with long maintenance intervals^18^. Fluorescent pH sensors can be highly sensitive, have high selectivity, excellent spatial and temporal resolution, and real-time in situ imaging^33,34^. Fluorescence-based optical oxygen sensors are already well-established in the oceanography industry^8,24^. New sensing materials need to be designed and explored to develop novel optical pH sensors. So far, only a few fluorophores have been identified that exhibit a significant change in the FL with changing pH^18^. To get around this problem, architectures revolving around the use of sensor films containing multiple chemical species have been proposed. For example, the dual-lifetime referencing (DLR) method has a well-known pH-sensing architecture^35–38^. The DLR architecture combines a pH-sensitive fluorophore with a long-lived luminescent reference, where the pH-sensitive indicator’s fluorescence properties vary with pH. The combined fluorescence response of the indicator pair is significantly longer and varies over a wider range than the pH-sensitive indicator alone, resulting in an easier optoelectronic readout^8,18,38^.

CDs are carbon-based nanoparticles with a size of  $<10$ nm, which, due to their diverse physiochemical properties, have attracted broad research in recent years^39–43^. CDs have recently emerged as a promising nanomaterial class that can be used to develop optical pH sensors^34^. Favourable traits include unique optical properties (high photostability, little to no photobleaching), affordability, low toxicity, and the abundance of exchangeable functional groups (e.g., amino, hydroxyl, carboxyl)^39,41,42,44–46^. The functional groups can be used to functionalise the surface of CDs, to alter their properties, including obtaining a brighter FI and prolonging the FL^47,48^. Several methods have been developed to synthesise CDs, including solvothermal, solid-phase, and microwave methods^22,48,49^. So far, most research on pH-sensitive fluorescent CDs has focused on the FI, while FL has received less attention^22^. Previous studies have resulted in syntheses of various CDs, with electronic structures that change with changes in pH, leading to a change in FI^22,34,50–57^, and emission wavelength^41,48,55,58–60^. Several CD-based pH sensors have been developed, including a general quantitative pH sensor^61^ as well as hybrid^62^ and ratiometric^63^ pH nano-sensors. Sensors based on pH indicators that show an FL change are lacking^64^. This is because all known pH FL indicators have lifetime decays in the ns domain, which was difficult to measure using compact and low-cost optoelectronics^18,26,27^ until recently, when cost-effective and sensitive optoelectronics started to be developed^65,66^. Affordable, frequency-domain-based techniques have been used to resolve FL to accuracies in the region of 0.01 ns^66^. There is a need to investigate potential new pH-sensitive fluorophores and CDs are attractive candidates for use in this type of sensor due to their favourable optical properties as mentioned above.

Theoretical studies have discussed, and proposed models on protonation and deprotonation, energy level changes, shell structure and proton transfer^48^. Still, a definite and acknowledged mechanism for CD behaviour has not been established^48,67^. Also, no well-defined and atomically precise CD structure has been reported. An in-depth analysis of the structure of the CDs and their optical properties is lacking^48,68,69^. Thus, exploring new CDs’ FI and FL properties is necessary, simultaneously providing comprehensive information when executing a study with CD as a pH sensing material.

Here, we present FI and FL measurements on five CDs for the pH range 5–9 using an excitation wavelength centred at 450 nm. CD04 is used as a reference sample using a synthesis published in the literature^22^. The pH range was chosen to cover those of interest for sensing applications in marine waters, where the pH is close to 8.2, and also applications in fresh water, where pH values are in the range of 6.5–9.0. Additionally, most waters have some capacity to resist pH change through the effects of the carbonate-buffer system. The wavelength of 450 nm was chosen since this was the nearest available wavelength for the sub-nanosecond pulse LED required for the FL measurements, to wavelengths normally used for fluorescence excitation in similar materials. The same excitation wavelength was used for the FI measurements. In order to investigate the choice of the emission filter wavelength on the fluorescence measurements, three different emission filters were used: 500/24 nm, 550/40 nm and 620/52 nm, where the numbers before and after the slash are the centre wavelength and full width at half maximum of the filter transmission, respectively. When compared, the three filters show no change in FL values, while the two latter are negatively affecting the FI. Absorption and excitation/emission spectra were obtained to determine the maximum FI wavelength. These spectra can be found in the Supplementary Information.

## Results

### Structural and morphological properties of CDs

**Figure 1:**
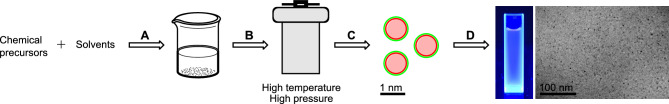
CDs synthesis: (**A**) chemical precursors are dissolved in a solvent, (**B**) the solution is transferred to an autoclave and heated at high temperature and pressure, (**C**) fluorescent CDs are formed, (**D**) analysis of CDs.

**Figure 2:**
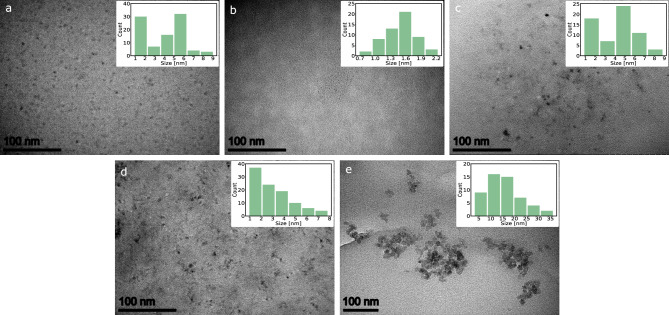
TEM images showing the size and morphology of the five CDs in the solid state (**a**) CD01, (**b**) CD02, (**c**) CD03, (**d**) CD04 and (**e**) CD05. The insets show the corresponding size distribution histograms of CDs.

**Figure 3:**
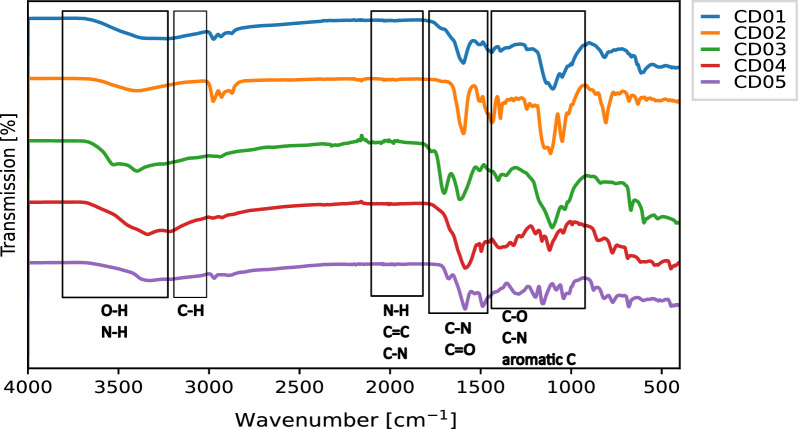
FTIR spectra of the five CDs in solid state (blue) CD01, (orange) CD02, (green) CD03, (red) CD04 and (purple) CD05. The boxed regions in the spectra show the characteristic spectral regions of group frequencies corresponding to sub-molecular groups of atoms found in the investigated CDs. From left: O–H (stretch), N–H (stretch), C–H (stretch), N–H (bend), C=C (stretch), C–N (bend), C–N (stretch), C=O (stretch), C–O (stretch), C–N (stretch) and aromatic C.

The five CDs have been synthesised using commercially available precursors by a straightforward hydro- or solvothermal procedure in an autoclave (Fig. 1), see Table 1 and Table S1 for an overview of characteristics and Methods section for the detailed synthesis method. Transmission electron microscopy (TEM) was used to establish the morphology and size distribution of the CDs (Fig. 2). Particles are monodispersed, except for particles in CD05, where they seem to cluster together. Most of the imaged particles have rough edges with non-uniform shapes and sizes. The size distributions of the CDs are shown in the insert (Fig. 2). The CDs’ diameters together with the main results are summarised in Table 1. The size distributions of the CDs are not Gaussian (except for CD02). CD01 and CD03 have two different most abundant particle diameters. The average diameter size for each CD is as follows: 4 ± 2 nm, 1.5 ± 0.3 nm, 4 ± 2 nm, 3 ± 2 nm and 15 ± 8 nm, for CD01, CD02, CD03, CD04 and CD05, respectively. The diameter of CD05 particles is bigger than that of the other CDs. To further investigate the structure of the CDs, Fourier transform infrared (FTIR) spectroscopy was used to identify CDs’ surface functional groups. Figure 3 show the FTIR spectra of the five CDs. The FTIR spectra are similar, with several molecular groups found across all spectra. All spectra contain an absorption band at 3400–3000 cm^−1^, which can be assigned to the –OH and –NH stretching vibrations. The peaks between 2980 and 2800 cm^−1^ can be attributed to C–H stretching vibrations. The peaks around 2000–1600 cm^−1^ can be assigned to the primary and secondary amides, C=O stretching vibrations and N–H bending vibrations. They can also be attributed to the aromatic C=C stretching vibrations. The bands corresponding to the C–O stretch can be found around 1400–1100 cm^−1^ (for CD01, CD02 and CD05), and the peaks corresponding to the C–N stretching vibrations can be found between 1460–1100 cm^−1^ for all CDs. Lastly, the peaks in the 1400–1300 cm^−1^ region can be assigned to the =C–H bending vibrations.

**Table 1:** Overview of the main results for the CDs, including the average diameter size [nm] obtained from TEM measurements, the average FI change [%] and FL change [ns], and [%] between pH 5 and 9 and corresponding R$^{2}$ values.

CD	Average size [nm]	FI change [%]	R$^{2}$ (FI)	FL change [ns]	FL change [%]	R$^{2}$ (FL)
01	4 ± 2	72	0.573	0.5	11	0.411
02	1.5 ± 0.3	69	0.981	0.4	9	0.817
03	4 ± 2	32	0.558	0.4	5	0.226
04	3 ± 2	87	0.532	0.5	17	0.974
05	15 ± 8	40	0.910	0.2	5	0.572

### PH dependent fluorescent response

**Figure 4:**
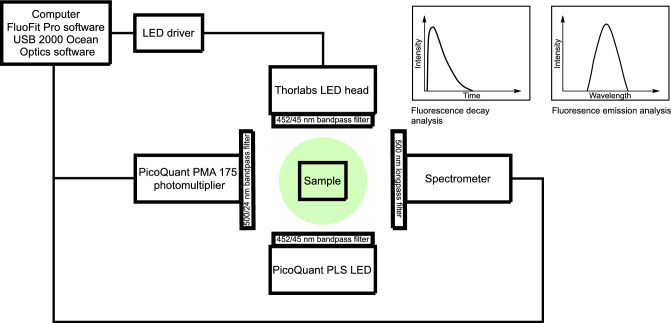
Schematic of the FI and FL experimental set-up.

In order to investigate the optical properties of CDs, fluorescence response was measured as a function of pH in the pH range 5–9. The CDs were dissolved in 100 mM Carmody buffer solutions to achieve the different pH environments. This buffer was chosen since it is a universal buffer that can be prepared to cover a wide range of pH values. Prior to measuring the fluorescence, the background noise was measured and subtracted. The FI and FL spectra were obtained from the experimental set-up shown in Fig. 4 and analysed using USB 2000 Ocean Optics and FluoFit Pro software for FI and FL measurements, respectively. The experimental setup is described in detail in the Methods section. A detailed analysis procedure can be found in the Supplementary Information section FI and FL measurements and analysis details. Additional measurements were performed on a second batch of CDs to examine how (1) temperature, (2) ionic strength of the buffer and (3) potential photobleaching, caused by prolonged exposure of the CDs to excitation light affect the fluorescence response of the indicator particles. The fluorescence response of CDs under the baseline conditions was compared with measurements at (1) a lower temperature (3 °C), (2) in buffers with 3.5% salt concentration, approximately matching that of seawater and (3) after being exposed to the excitation source for at least 52 hours. The main results are summarised in Table 1, and two extended tables can be found in the Supplementary Information, section FI and FL values.

#### FI

**Figure 5:**
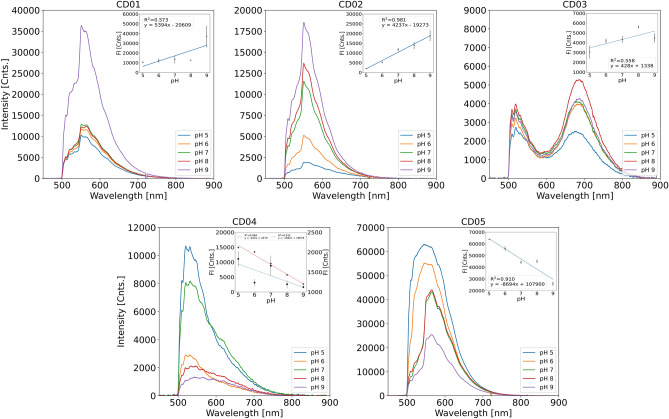
FI spectra of the five CDs (0.02 mg $\cdot$ mL$^{-1}$) dissolved in 100 mM Carmody buffer solutions adjusted to pH 5–9. The insets show the change in maximum peak intensity as a function of pH. A trend-line has been fitted through the data points for each CD. Standard errors of the slopes are as following: 2689 (CD01), 339 (CD02), 220 (CD03), 1053 (CD04) and 1502 (CD05). For the CD04 inset, the FI data from the literature has been plotted (triangles) and a trend-line fitted (red). Standard error of the red trend-line is 18.

Figure 5 shows the FI spectra of all five CDs. Each CD shows a reduction in FI as a function of pH. The insets in Fig. 5 show the change in maximum peak intensity as a function of pH. For CD01, CD02 and CD03, the fluorescence gets brighter with increasing pH. Between pH 9 and 5, the FI is reduced by 72%, 69%, and 32% for CD01, CD02 and CD03, respectively. An opposite trend is observed for CD04 and CD05. Here is an 87% and 40% reduction in intensity between pH 5 and 9 for CD04 and CD05, respectively. A trend-line and its equivalent R$^{2}$ values have been fitted to the data. CD02 and CD05 show the best linearity with R$^{2}$ values of 0.981 and 0.910, respectively. The linearity of the other CDs is as follows: 0.573 for CD01, 0.558 for CD03 and 0.532 for CD04. Shortly summarised, CD01 and CD04 exhibit the greatest change in FI across pH 5–9. Both the change in temperature and the ionic strength of the buffer affect the CDs in different ways. In general, the decreased temperature increased the fluorescence response. On average, the FI increased by 9%, 19%, 71% and 5% for CD01, CD02, CD03 and CD05, respectively. CD04 shows a slight decrease of 8% in the FI. In salt buffers, most of the CDs show an increase in the fluorescence response. On average, the FI changed by 3%, −24%, 36%, 188% and 4% for CD01, CD02, CD03, CD04 and CD05, respectively. The pro-longed excitation of the CDs did not affect the FI of the CDs, except for CD03 and CD04, where the FI changed by −30% and 24%, respectively.

#### FL

**Figure 6:**
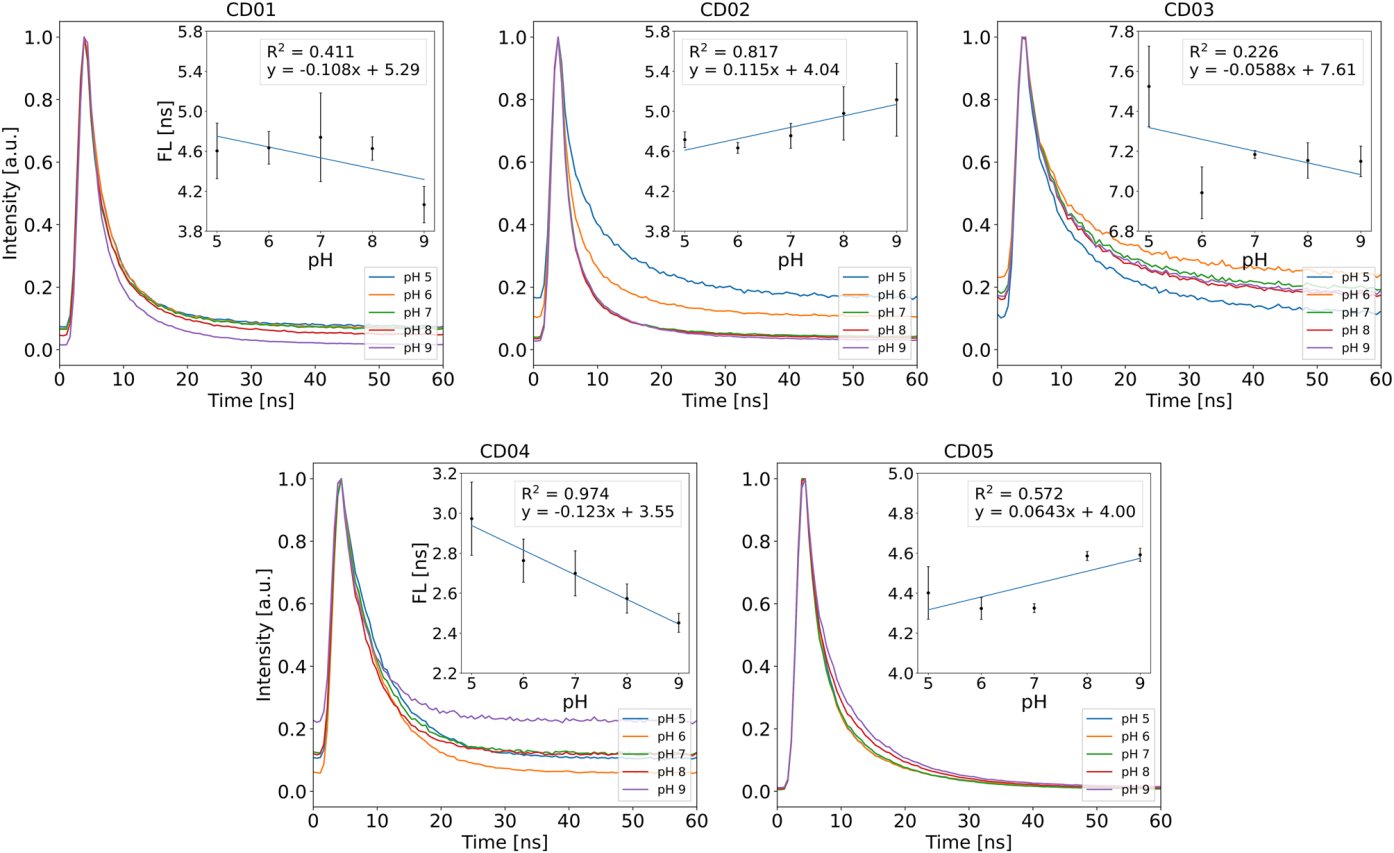
Time-resolved fluorescence decay of the five CDs (0.02 mg $\cdot$ mL$^{-1}$) dissolved in 100 mM Carmody buffer solutions adjusted to pH 5–9. The insets show the calculated FL as a function of pH. A trend-line has been fitted through the data points for each CD. Standard errors of the slopes are as following: 0.0748 (CD01), 0.0313 (CD02), 0.0629 (CD03), 0.0116 (CD04) and 0.0322 (CD05).

Figure 6 shows FL at different pH for all five CDs. Out of the five CDs, CD03 has the longest FL of around 7 ns. CD01 and CD02 emit fluorescence with a lifetime of around 5 ns. CD04 and CD05 have FL of 2.7 and 4.4 ns, respectively. The five CDs show different responses of FL as a function of pH. CD01 and CD02 show opposite trends. In acidic environments (pH 5–7), the FL of CD01 and CD02 remains rather constant. In basic environments, the FL of CD01 decreases from 4.7 ± 0.4 ns at pH 7 to 4.1 ± 0.2 ns at pH 9. Simultaneously, the FL of CD02 increases from 4.8 ± 0.1 ns at pH 7 to 5.1 ± 0.4 ns at pH 9. A similar trend can be seen when comparing CD04 and CD05. FL of CD04 decreases with increasing pH from 3.0 ns ± 0.2 ns at pH 5 to 2.5 ± 0.1 ns at pH 9, and CD05’s FL increases with increasing pH from 4.4 ns ± 0.1 ns at pH 5 to 4.6 ± 0.1 ns at pH 9. Similarly to CD01 and CD04, the FL of CD03 decreases with increasing pH and changes from 7.5 ± 0.2 ns at pH 5 to 7.1 ± 0.1 ns at pH 9. Briefly, the changes in FL between pH 5-9 are as follows: 0.5 ns (11%), 0.4 ns (9%), 0.4 ns (5%), 0.5 ns (17%) and 0.2 ns (5%) for CD01, CD02, CD03, CD04 and CD05, respectively. To inspect the data trends, a trend-line and its equivalent R$^{2}$ values have been fitted to the obtained data. CD02 and CD04 show the best linearity with R$^{2}$ values of 0.817 and 0.974, respectively. The linearity of the other CDs is lower, but an increasing FL trend with pH between the obtained data for CD05 and a decreasing trend for CD01 and CD03 are observed. Shortly summarised, CD04, CD01 and CD02 exhibit the greatest change in FL across pH 5-9, with CD04 and CD02 showing the greatest linearity. At low temperature, CD01 and CD04 show an average increase in FL of 3% and 7%, respectively, over the range of pHs tested, increasing with increasing pH. CD02 shows a decrease of FL by 2%, with FL falling well within the uncertainty of the measurement. CD03 shows on average no change of FL. CD05 shows a decrease of 9% on average. In salt buffers, CD01 and CD03 show a slight increase in FL of 2%. CD02 remains unchanged on the average, while CD04 and CD05 show an average increase in FL by 23% and 8%, respectively. The pro-longed excitation of the CDs slightly increased the FL of the CDs by up to 6% for CD02 and 4% for CD03 and CD04.

## Discussion

The pH-dependent fluorescence response of the five CDs in the 5–9 pH range was investigated. CD02 and CD05 display linear relationships between FI and pH. Thus, it can be concluded that the FI of the two CDs is sensitive to pH changes. The reason for different FI responses to pH is presumably the differences in the size of the CDs and the fact that the surfaces of CDs have different functional groups, so-called surface-state fluorescence^70^. The differences in the intensity and placement of the FTIR peaks confirm the presence of different surface-states. Depending on the pH environment, the abundant functional groups, including carboxylic acids and amide groups, can either protonate or deprotonate to various degrees^48,71^. The changes in the complex surface state of the particle affect the proton and electron transfer between the particle and the buffer. CD01, CD02 and CD3 show an increasing FI trend with increasing pH. This trend can be ascribed to the deprotonation of the carboxyl groups abundant on the CDs surface and can be attributed to the fact that sulfuric acid and citric acid were used in the synthesis of the three CDs. It has been previously reported that CDs with abundant carboxyl groups show this behaviour due to the deprotonation of the groups and formation of a delocalised $\pi$-bond, enhancement of n electrons and shift in the Fermi level, causing charging/electrostatic doping of the CDs^48,60,70,72^. On the other hand, CD04 and CD05 show a decreasing trend in FI with increasing pH. This behaviour can be attributed to the proton transfer from a protonated nitrogen to the conjugated carbon structure^48^, as previously shown in the literature^57^. It should be noted that the syntheses of CD04 and CD05 include nitrogen-rich precursors, and the decreasing trend in FI can be attributed to the nature of these. CDs are still a novel field of research, and a lot of uncertainty is associated with understanding the mechanism of pH-sensitive fluorescence. Up till now, the research has been strongly focused on the deprotonation and protonation of the carboxyl and amide groups^73^, as discussed above. Still, several other mechanisms for pH quenching in acidic environments also have been put forward, including the change of the energy level, aggregation of the CDs and pH-induced electron transfer^48,73^.

CD05 exhibits the strongest FI out of the five CDs, almost six times more intense than CD04. When comparing CD05 to CD04, the addition of phloroglucinol and DMF in the synthesis of CD05 resulted in an increase in both the FI (475 % increase) and the FL (7 % increase). CD05 is better resolved in regards to FI as a function of pH, having better linearity between FI and pH, R$^{2}$ = 0.532 (CD04) to 0.910 (CD05). However, the linearity for FL decreased from R$^{2}$ = 0.974 (CD04) to 0.572 (CD05). A similar situation occurs between CD01 and CD02. The main difference in synthesising these two CDs is that toluene was added to the crude product after the synthesis and the toluene fraction was later removed and evaporated, resulting in CD02. The remaining aqueous layer was dried and resulted in CD01. The difference in the synthesis resulted in a decreased FI (−98 %) for CD02, but improved linearity of R$^{2}$ = 0.981, compared to 0.573 for CD01. The overall FL is longer for CD02 (7% increase), additionally having better linearity (R$^{2}$ = 0.817) compared to CD01 (R$^{2}$ = 0.411). Thus, it can be concluded that the use of toluene positively affected the fluorescence response to pH. The fact that CD03 has the longest FL, followed by CD01 and CD02, suggests that the disperse blue 1 dye as a chemical precursor in the synthesis of CDs has a positive effect on the FL. The FI spectrum of CD03 has a distinctive shape compared to the other CDs. It has two peaks, one at 520 nm and the second at 690 nm. The second peak at the longer wavelength is most likely due to another fluorescence process. It has previously been reported that CDs prepared by solvothermal synthesis with the assistance of ammonium fluoride tend to have red-shifted FI^74^. The red-shift occurs due to fluorine having a strong electron-withdrawing tendency, which decreases the energy level between the highest occupied molecular orbital and the lowest occupied molecular orbital^74–76^.

When comparing the size of the CDs with the FI measurements, it is possible to see that the CDs with the smallest (CD02) and biggest (CD05) average sizes exhibit the best linearity between FI and pH. No trend based on the size of the CDs could be identified for the fluorescence response. Above 10 nm, carbon nanoparticles start to possess more physical state attributes and lose their chemical state properties (i.e., fluorescence)^39^. Keeping particles below 10 nm is therefore important. However, particle size is not the dominant determinant of the five CDs’ fluorescence, with molecular states and surface defect states being responsible for fluorescence. A smaller size-distribution is always beneficial so that all particles have roughly the same composition and behaviour.

The response of optical pH indicators can generally be considered linear within a relatively narrow pH range about the indicator pKa. The response rapidly decreases for pH values above and below this region. Since CD02 and CD04 show high linearity between pH 5–9, this implies that the pKa of these indicators most likely lies towards the centre of this range. Using the same argument, the reduced response linearity to pH of CD01, CD03 and CD05 implies that the pKa of these indicators may lie towards the extremes or outside the tested pH range. In the response, non-linearity may also be caused if multiple pH indicators with different pKa are present in the CDs. The uncertainty of the FL measurements can also be attributed to the background noise and low signal-to-noise ratio, which leads to significant uncertainty in the fit of the fluorescence decay. Variations in the number of CDs in each sample can also affect the measurements and cause more significant deviations between individual measurements.

CD04 was synthesised by adapting a published synthesis of N-CDs^22^. CD04 was used as a reference for the four CDs. CD04 has an average diameter size of 3 nm, while the N-CDs are reported with an average diameter of 5 nm. The FTIR spectra of the two CDs both show the characteristic peaks of the aforementioned functional groups, including C=O stretching vibrations in COOH groups, stretching of C–N and C=C, and C–O and aromatic C stretching vibrations at 1500–1100 cm^−1^. The FI response of the CDs shows a decreasing trend with increasing pH. CD04 exhibit worse linearity (R$^{2}$ = 0.532) than N-CDs (R$^{2}$ = 0.984). The reported average FL is longer for N-CDs (4.8 ns) than for CD04 (2.7 ns). The change in the FL is 0.15 ns for N-CDs from pH 6.2 to 8.6 and 0.19 ns from pH 6–8 for CD04. Both CDs show a decreasing FL trend with increasing pH. The differences in sizes of the two CDs can be attributed to the adaptations made in the synthesis method. CD04 was synthesised using 1 g of m-PD and 40 mL of water, as opposed to 100 mg m-PD and 10 mL water for N-CDs. Still, the CDs follow the same fluorescence trends with changing pH.

The investigations show that the fluorescence response for the CDs varied slightly between batches. This is most likely due to the difficulty of exactly replicating the bottom-up synthesis process, leading to small variations in the relative abundance of the different precursors in the CDs. However, the reported trends found are the same.

The environmental factors on the CDs have been examined. After prolonged excitation, the CDs show little to no change in fluorescence response, proving the low photo-bleaching properties of CDs, with exception of CD03 and CD04. The fact that FI is more affected by photo-bleaching shows the relative robustness of the FL characteristic. From these measurements, it can also be seen that the FL of CD01, CD02, and CD03 are not significantly affected by either the temperature change or the ionic strength of the buffer, showing the stability and resilience of these carbon nano-particles to changing environmental conditions. The change in the fluorescence response for CD04 and CD05 when measured in salt buffer and at low temperature, can indicate that regardless of the general robustness of CDs, CDs and their fluorescence response can be altered by the precursor choice. Overall, the CDs follow the general trend of increasing emission intensity with decreasing temperature^77^. An exception here seems to be CD04, which shows a slight decrease of 8%. However, when measurement uncertainty is taken into account, it can be observed that also this CD follows the general trend. The results from these measurements show that in order to use any of these CDs, an assessment needs to be made of which environmental factors affect the CDs, and ensure that any sensors incorporating the CDs are calibrated against these parameters in order to maintain accuracy.

## Conclusion and future perspectives

In this paper we present five different CDs and the corresponding FI and FL measurements as a function of pH. In summary, CD02 and CD05 show the best linear relationship between pH and FI. CD01, CD02 and CD03 exhibit brighter fluorescence with increasing pH, the opposite trend is found for CD04 and CD05. CD04 shows the clearest monotonic change in FL with pH, while CD03 has the longest FL of around 7 ns. As is, none of the CDs shows strong enough FL dependency in order to develop a FL-based pH sensor. The composition and abundance of functional groups at the surface of the CDs is the main factor affecting the fluorescence response. While the synthesis of the CDs should be further explored, the particle size does not crucially affect the fluorescence response. Disperse blue 1 dye has been shown to result in longer FL when used as a precursor. The CDs were found to be somewhat affected by temperature and ionic strength changes of the buffers, however, the CDs have shown not to be significantly affected by photo-bleaching. This is particularly important for measurement applications covering longer periods of time. More experiments should be performed to improve the fluorescence response of the CDs by altering the functional groups on the surface of the dots, by appropriate modifications of the present CD’s synthesis with respect to the choice of precursors, their masses and concentrations, as well as time and temperature of the syntheses. CDs fluorescence response is a novel field, and more research and data are needed.

## Methods

### Materials

Disperse blue 1 dye, phloroglucinol, ethanol, sulfuric acid, toluene, m-PD, DMF, monohydrate citric acid, and ammonium fluoride were obtained from Sigma Aldrich. Ultrapure water (Milli-Q) was used in the synthesis of the CDs.

### Synthesis and purification of CDs

#### CD01 and CD02

Briefly, 0.3 g of disperse blue 1 dye and 0.7 g of phloroglucinol were dissolved in 40 mL ethanol containing 7.5 % sulfuric acid. The solution was transferred to a Teflon-lined hydrothermal vessel and heated at 185 °C for 3.5 h. The resulting solution was cooled at ambient temperature and re-suspended in 100 mL of toluene: water (1:1 v/v). The toluene fraction was removed, and solvent was evaporated within a fume hood. This fraction was dissolved in ethanol and subjected to dialysis (MWCO 3500) using ultrapure water for 72 h before being freeze-dried. This sample was named CD02. The water-soluble fraction was initially dried within a fume hood and was re-suspended in 10 mL ethanol before being diluted in water to 100 mL. This solution was filtered through a syringe filter (0.2 $\upmu$m) before being dialysed (MWCO 3500) against ultrapure water for 72 h. The resulting solution was freeze-dried and was named CD01.

#### CD03

0.3 g of disperse blue 1 dye, 0.4 g citric acid, and 0.1 g ammonium fluoride was made up to 30 mL in DMF. This solution (ammonium fluoride not dissolved) was added to a Teflon-lined hydrothermal vessel and heated at 180 °C for 3 h. The resulting solution was dried in a fume hood on a plastic plate before being re-suspended in water and subjected to dialysis (MWCO 3500) against ultrapure water for 72 h. The purified particles were freeze-dried.

#### CD04

1 g of m-PD was dissolved in 40 mL of water. The solution was transferred to a Teflon-lined hydrothermal vessel and heated at 180 °C for 3 h. The resulting solution was cooled at ambient temperature and subjected to dialysis (MWCO 3500) using ultrapure water for 72 h before being freeze-dried. The procedure was adapted from the literature^22^.

#### CD05

1 g of m-PD and 1 g of phloroglucinol were dissolved in 40 mL DMF containing 0.6% sulphuric acid. The solution was added to a Teflon-lined hydrothermal vessel and heated at 185 °C for 3.5 h. The resulting solution was subjected to dialysis (MWCO 3500) against ultrapure water for 72 h before being freeze-dried.

Ethanol was added to all five CDs to obtain a stock solution with a concentration of 1 mg mL$^{-1}$. The measured samples consisted of 20 uL CDs stock solution and 980 uL Carmody buffer in the pH range 5–9. Details on the buffer synthesis can be found in the Supplementary Information section Carmody buffer solutions. The concentration of CDs in all measurements was 0.02 mg mL$^{-1}$.

### Fluorescence measurements

#### FI

A continuous wave of white Thorlabs LED with a 452 nm spectral filter was used to illuminate the samples. USB 2000 Ocean Optics spectrometer fitted with a 500 nm longpass filter was used to collect and analyse the resulting fluorescence.

#### FL

A PicoQuant PLS light emitting diode with emission centred at 450 nm was used to stimulate fluorescence excitation to obtain the FL of the dots. The fluorescence was collected by a PicoQuant PMA 175 photomultiplier fitted with a 24 nm bandpass spectral filter centred at 500 nm. The response was digitised using TimeHarp 260 Nano. The reported FL values were determined in FluoFit Pro software by fitting exponential curves to the resulting fluorescence decay data.

### Characterisation of CDs

TEM images were captured using a Hitachi HT7800 microscope operated at an accelerating voltage of 80 kV. All CDs were added to a 50% ethanol solution and sonicated for 20 min (except for CD02) prior to being drop casted onto carbon/copper grids. CD02 sample was added to 100% ethanol prior to drop casting. Grids were dried at ambient temperature for 12 h prior to imaging. FTIR spectra were obtained on a Nicolet iS50R FTIR spectrometer with the diamond ATR crystal technique, with 32 scans/sample and resolution of 4.0 cm$^{-1}$.

## Statistical analysis

All data are presented as the mean ± standard error of the mean (s.e.) of three independent experiments. See Supplementary Information section FI and FL values for more details.

## Supplementary Information

Supplementary Information.

## Data Availability

All relevant data can be found in the article and its Supplementary Information files. The datasets used and analysed in this study are available from the corresponding author on request.
