# Supplementary material for: Fluorescence intensity and fluorescence lifetime measurements of various carbon dots as a function of pH
Source: Sci Rep. 2023 Jun 30;13:10660. doi: 10.1038/s41598-023-37578-z (PMC10313676; doi:10.1038/s41598-023-37578-z)
Supplement: Supplementary file 1 — Supplementary Information. [file 41598_2023_37578_MOESM1_ESM.pdf]

Supplementary Information

Fluorescence Intensity and Fluorescence Lifetime Measurements of Various Carbon Dots as a Function of pH

Wiktoria K. Szapoczka[1,*], Adam L. Truskewycz[2], Tore Skodvin[3], Bodil Holst[1], and Peter J. Thomas[4]

[1]University of Bergen, Department of Physics and Technology, Bergen, 5007, Norway
[2]University of Bergen, Department of Biomedicine, Bergen, 5009, Norway
[3]University of Bergen, Department of Chemistry, Bergen, 5007, Norway
[4]NORCE Norwegian Research Centre AS, Bergen, 5008, Norway
[*]Wiktoria.Szapoczka@uib.no

Carmody buffer solutions

Buffer solutions were prepared following the recipe by W.R. Carmody[1]. Shortly summarised, two solutions, A (boric acid, 0.2 M, citric acid, 0.05 M) and B (tertiary sodium phosphate, 0.1 M), were prepared and mixed with Ultrapure water (Milli-Q) following the ratios in Table S1. This resulted in a 0.1 M Carmody buffer series in the pH range of 5-9.

Table S1. Volumes of solution A, B and Ultrapure water (Mili-Q) used to prepare 50 mL of the buffer solutions.

pH	Solution A [mL]	Solution B [mL]	Ultrapure water [mL]	Final volume [mL]
5	16,71	8,23	25,06	50
6	15,65	10,88	23,47	50
7	14,20	14,49	21,31	50
8	12,98	17,56	19,47	50
9	11,37	21,58	17,05	50

Excitation and emission spectra

Fluorescence measurements were conducted on a Tecan Spark 10M Multimode Plate Reader within black 96-well plates (Costar). The excitation and emission fluorescence profiles of CDs were generated using aqueous resuspensions of freeze-dried treatment (1%) with 20 nm incremental excitation steps between 300 and 700 nm.

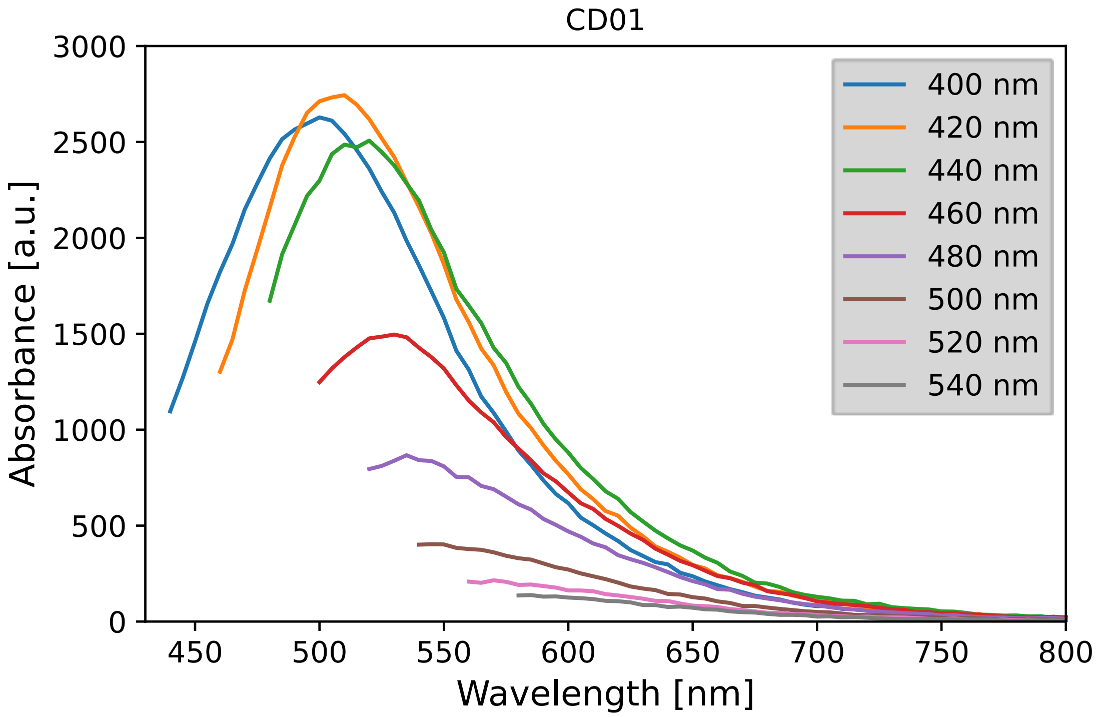

Figure S1. Excitation and emission spectra of CD01 aqueous resuspensions of freeze-dried treatment (1%) with 20 nm incremental excitation steps between 300 and 700 nm.

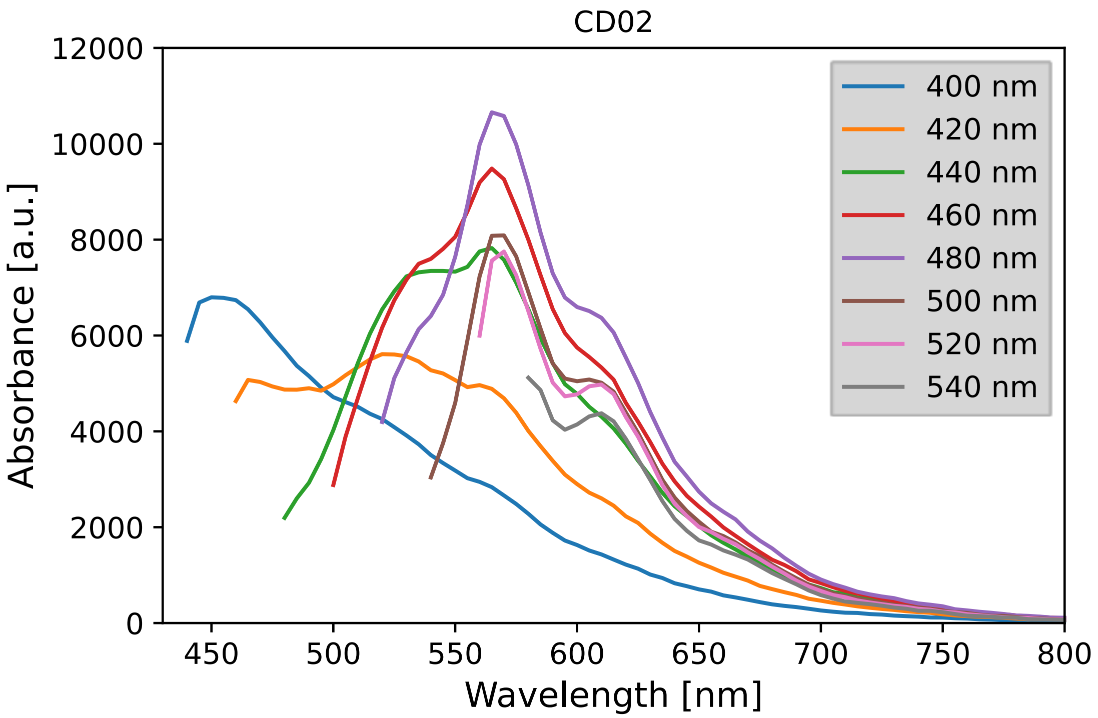

Figure S2. Excitation and emission spectra of CD02 aqueous resuspensions of freeze-dried treatment (1%) with 20 nm incremental excitation steps between 300 and 700 nm.

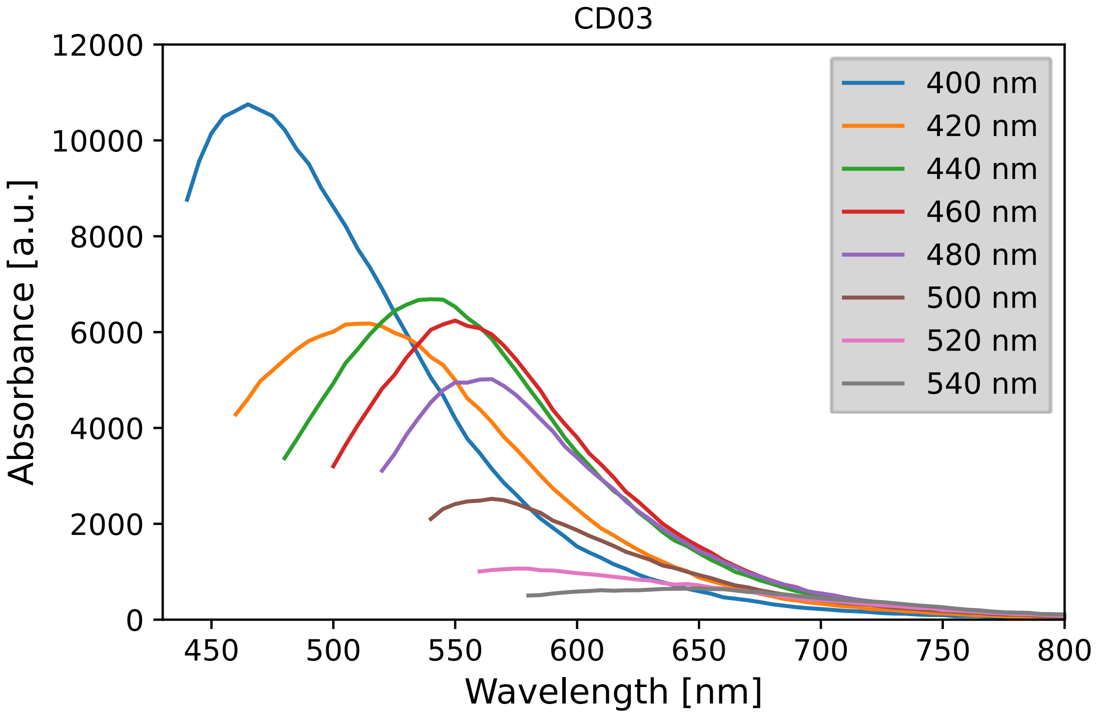

Figure S3. Excitation and emission spectra of CD03 aqueous resuspensions of freeze-dried treatment (1%) with 20 nm incremental excitation steps between 300 and 700 nm.

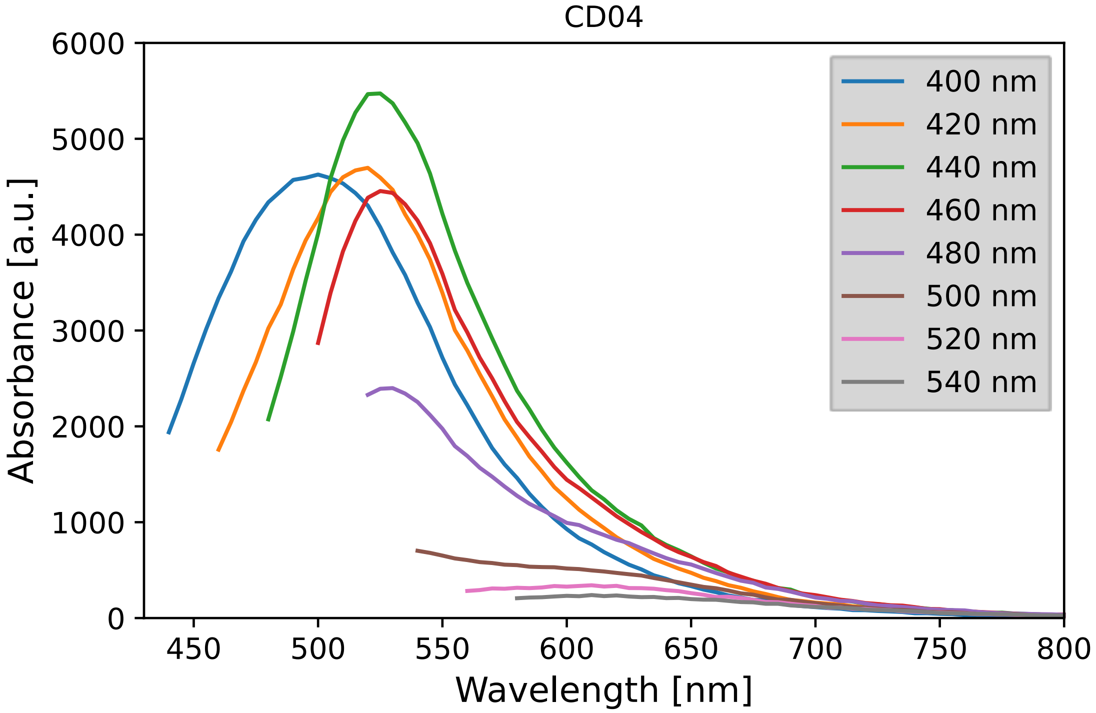

Figure S4. Excitation and emission spectra of CD04 aqueous resuspensions of freeze-dried treatment (1%) with 20 nm incremental excitation steps between 300 and 700 nm.

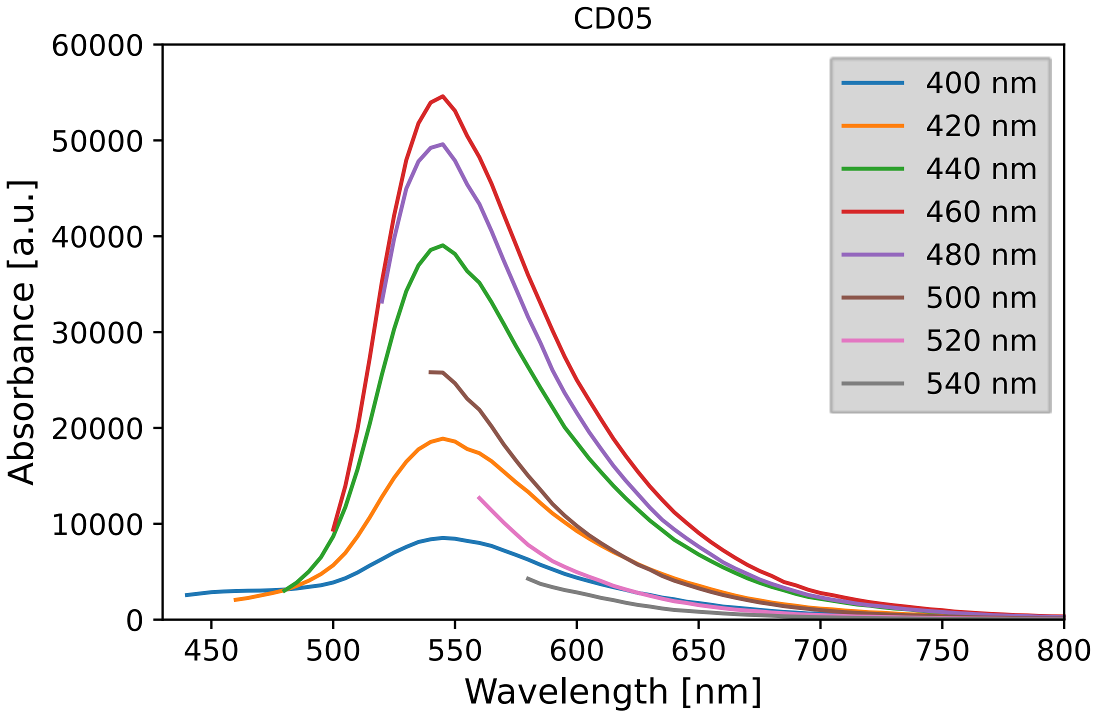

Figure S5. Excitation and emission spectra of CD05 aqueous resuspensions of freeze-dried treatment (1%) with 20 nm incremental excitation steps between 300 and 700 nm.

Absorption spectra

UV/Vis absorption measurements were conducted on an UV-1800 Shimadzu UV Spectrophotometer and recorded by UVProbe 2.50.

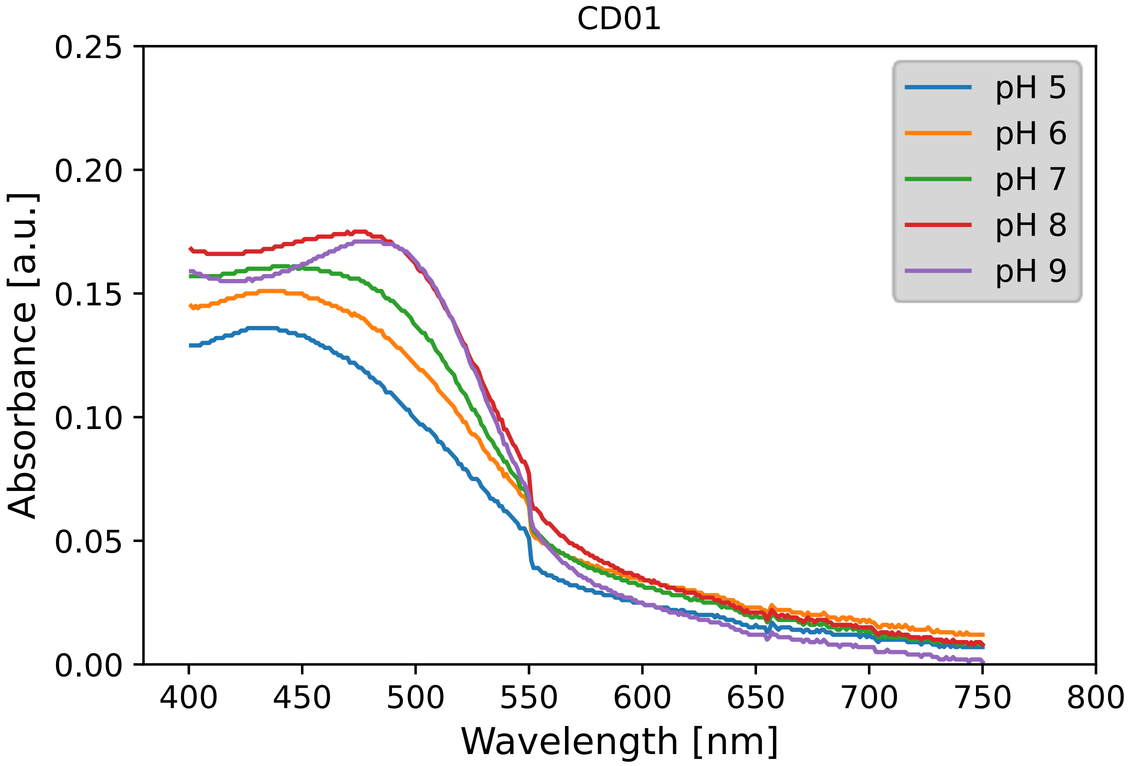

Figure S6. UV-Vis absorption spectra of CD01 measured at different pHs ranging from 5 to 9.

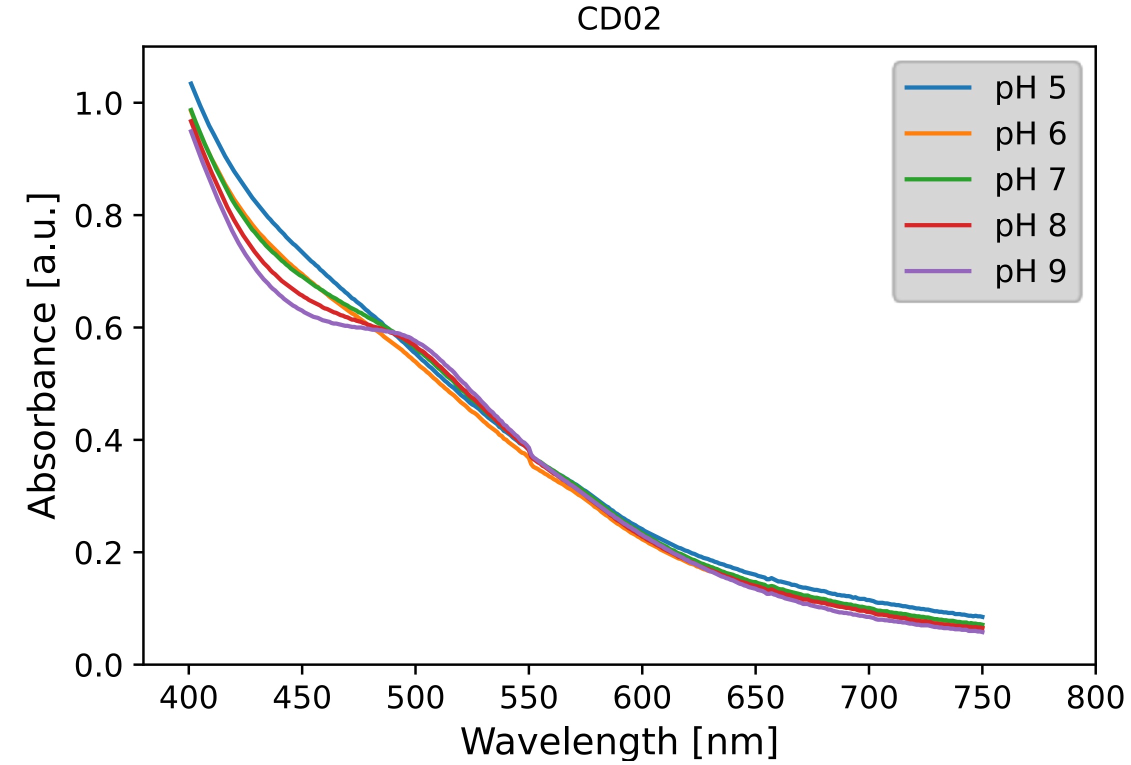

Figure S7. UV-Vis absorption spectra of CD02 measured at different pHs ranging from 5 to 9.

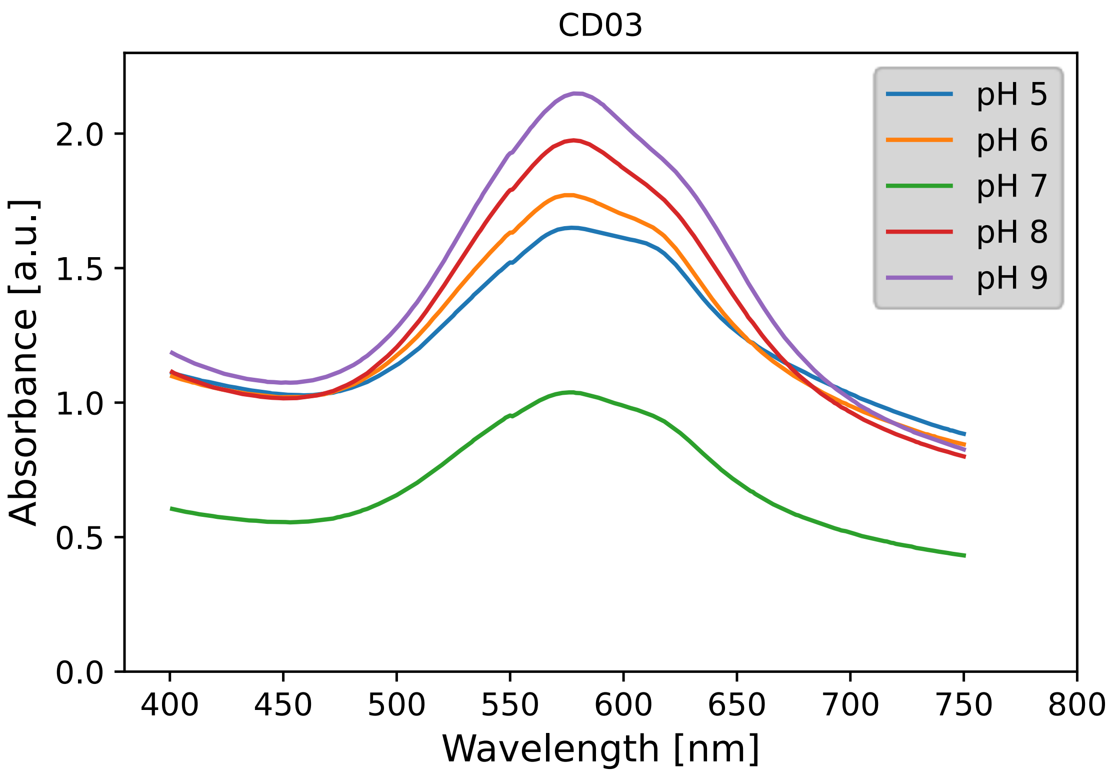

Figure S8. UV-Vis absorption spectra of CD03 measured at different pHs ranging from 5 to 9.

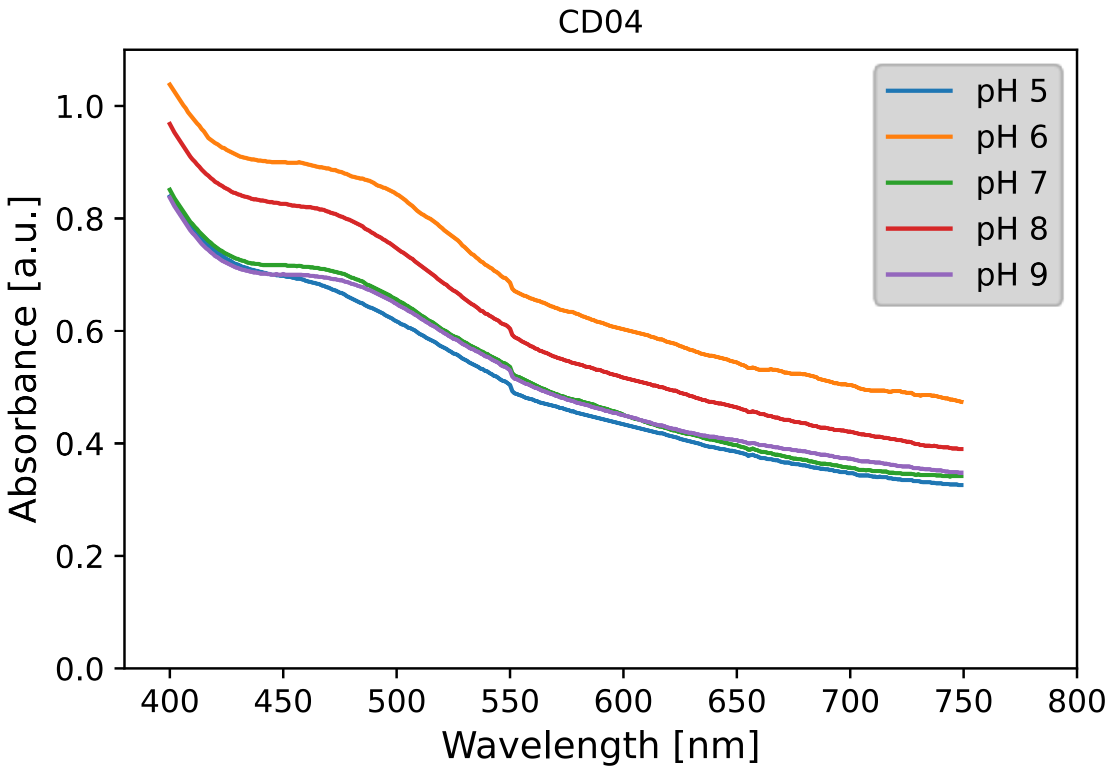

Figure S9. UV-Vis absorption spectra of CD04 measured at different pHs ranging from 5 to 9.

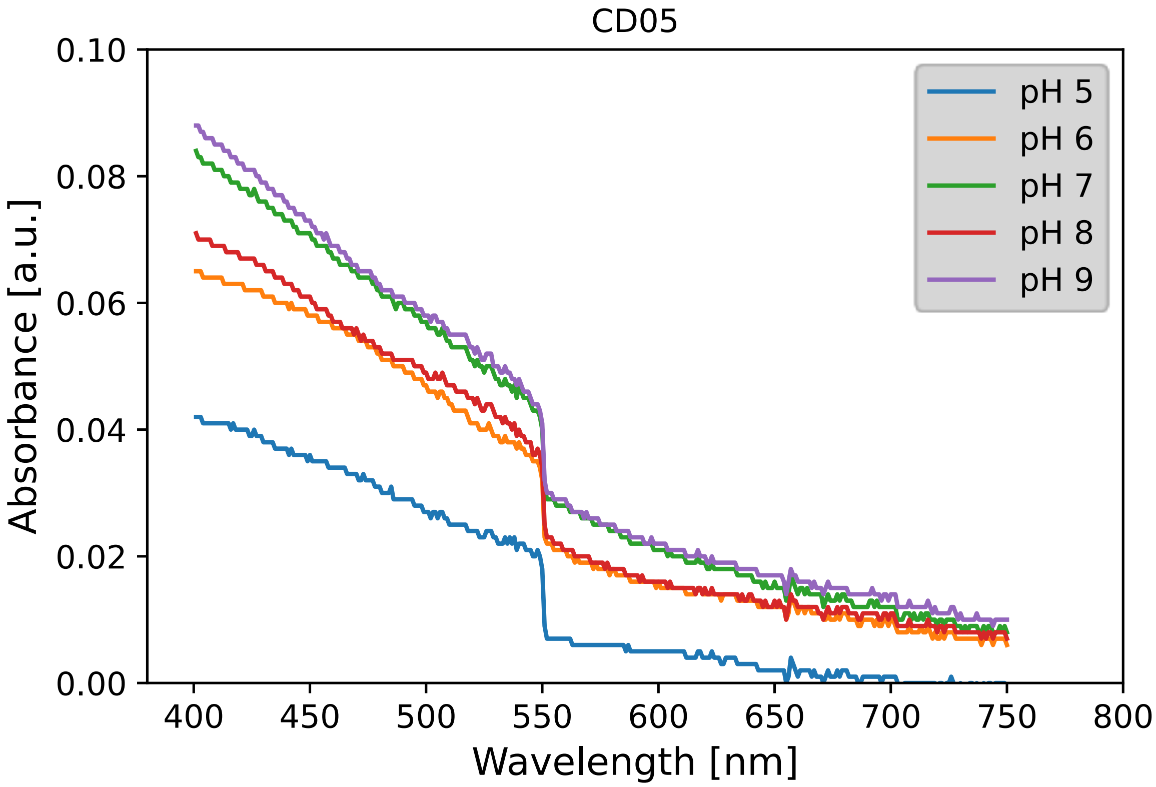

Figure S10. UV-Vis absorption spectra of CD05 measured at different pHs ranging from 5 to 9.

FI and FL measurements and analysis details

Tables S2 and S3 summarise the FI and FL measurement and analysis settings used to obtain data for this study.

Table S2. Measurement and analysis specifications for FI values of the CDs.

Measurement software	Ocean Optics	
Illumination source	ThorLabs LED	452 nm spectral filter
	Intensity	Maximum
Spectrometer	USB 2000 Ocean Optics	500 nm longpass filter
Analysis software	Ocean Optics	
	Number of independent experiments	Three
	Integration time	10 000 ms

Table S3. Measurement and analysis specifications for FL values of the CDs.

Illumination source	PicoQuant PDL 800-B PLS light emitting diode	centred at 450 nm	
	Intensity	10 (maximum)	
Photomultiplier	PicoQuant PMA 175	500/24 nm bandpass filter	
Measurement software	TimeHarp 260 Nano S/N 1031843		
	Rep. frequency	8	
	Trigger out	1 us	
	Sync Input	Edge	0
		Divider	None
		Level [mV]	51
		Offset [ps]	10000
	Inp. Chan. 1	Edge	0
		Level [mV]	50
		Offset [ps]	0
	Inp. Chan. 2	Edge	0
		Level [mV]	50
		Offset [ps]	0
	Acquisition	Resol. [ns]	0,25
		Offset [ns]	20
		Time [s]	60
		Trc/Block	0
		Mode	OSC
		Stop at [cnt]	4294967295
Analysis software	FluoFit Pro		
	Exp. [Tailfit]		
	Number of Exponentials	2	
	Range	Decay	
	Min	3	
	Max	160	

FI and FL values

Values obtained in the analysis of FI and FL of CDs. Values in Table S4 correspond to the results shown in Figures 5 and 6 in the main text. The values were obtained by calculating the mean of three independent experiments and are presented with the standard error (s.e.) of the mean.

Table S4. Fluorescence response of the first batch of CDs in Carmody buffers. FI [cnts.], FL [ns] and standard deviation of the FI [cnts.] and FL [ns] values for the CDs for the pH range 5-9.

CD	pH	FI [cnts.]	s.d. FI [cnts.]	FL [ns]	s.d. FL [ns]
1	5	10535	654	4,603	0,3
	6	12076	1837	4,634	0,2
	7	13289	3508	4,739	0,4
	8	12636	378	4,627	0,1
	9	37228	10563	4,066	0,2
2	5	2053	119	4,715	0,1
	6	5237	355	4,633	0,1
	7	11833	724	4,755	0,1
	8	13916	1648	4,979	0,3
	9	18900	2538	5,115	0,4
3	5	3058	630	7,524	0,2
	6	4176	209	6,992	0,1
	7	4354	311	7,184	0,1
	8	5601	118	7,154	0,1
	9	4485	425	7,149	0,1
4	5	11109	2498	2,973	0,2
	6	3141	1156	2,763	0,1
	7	8798	3327	2,699	0,1
	8	2605	1217	2,573	0,1
	9	1650	344	2,451	0,1
5	5	63861	183	4,401	0,1
	6	56049	1538	4,324	0,1
	7	44324	1144	4,325	0,1
	8	45096	1234	4,586	0,1
	9	25866	1601	4,592	0,1

Table S5. Fluorescence response of the second batch of CDs in Carmody buffers, Carmody buffers with 3.5% salt, at low temperature and after photobleaching. FI [cnts.], FL [ns] and standard deviation of the FI [cnts.] and FL [ns] values for the CDs for the pH range 5-9.

		Carbon dots in Carmody buffers, second batch				Ionic strength (3,5%)				Low temperature (3°C)				Photobleaching	
CD	pH	FI [cnts.]	s.d. FI [cnts.]	FL [ns]	s.d. FL [ns]	FI [cnts.]	s.d. FI [cnts.]	FL [ns]	s.d. FL [ns]	FI [cnts.]	s.d. FI [cnts.]	FL [ns]	s.d. FL [ns]	FI [cnts.]	FL [ns]
1	5	4200	740	4,004	0,2	5923	1019	4,067	0,1	6094	651	3,712	0,1	-	-
	6	9666	103	3,384	0,1	9634	1117	3,502	0,1	9700	1831	3,425	0,1	-	-
	7	20363	1257	3,034	0,1	19507	1168	3,151	0,1	19536	1283	3,193	0,1	21764	3,058
	8	47644	2110	3,083	0,1	33428	1093	3,087	0,1	41553	2089	3,302	0,1	-	-
	9	52569	1928	3,279	0,1	57948	1908	3,301	0,1	61985	656	3,598	0,1	-	-
2	5	4257	42	3,223	0,1	3211	90	3,252	0,1	4873	321	3,241	0,1	-	-
	6	5873	277	2,945	0,1	4295	159	3,002	0,1	7925	188	2,947	0,1	-	-
	7	11422	110	2,936	0,1	8682	259	2,903	0,1	12546	574	2,860	0,1	11412	3,117
	8	22888	956	3,114	0,1	16424	402	3,099	0,1	25498	2225	3,006	0,1	-	-
	9	34340	760	3,290	0,1	28406	854	3,277	0,1	41842	1877	3,190	0,1	-	-
3	5	2818	180	6,644	0,1	2176	1095	6,688	0,1	5617	476	6,603	0,1	-	-
	6	1365	438	6,499	0,1	3203	253	6,637	0,1	3857	1795	6,380	0,1	-	-
	7	2238	160	6,334	0,1	3049	199	6,479	0,1	2617	111	6,343	0,1	1566	6,583
	8	2453	1234	6,250	0,1	3523	160	6,363	0,1	3370	1098	6,248	0,1	-	-
	9	3901	347	6,212	0,1	3385	101	6,271	0,1	4723	222	6,313	0,1	-	-

4	5	10833	630	3,518	0,1	22936	1952	4,000	0,1	6766	351	3,810	0,1	-	-
	6	17871	3247	3,284	0,1	27789	5684	3,858	0,1	16290	2510	3,567	0,1	-	-
	7	10738	3882	2,971	0,1	30244	2415	3,678	0,1	9593	1330	3,178	0,1	13297	3,079
	8	9733	3691	2,800	0,1	27025	4026	3,676	0,1	9702	3161	3,080	0,1	-	-
	9	6145	1753	3,206	0,1	31572	2276	4,035	0,1	7107	2146	3,177	0,1	-	-
5	5	28750	1044	5,063	0,1	29214	1368	5,494	0,1	15315	204	4,653	0,1	-	-
	6	20201	1431	4,663	0,1	21741	1740	5,357	0,1	11840	719	4,276	0,1	-	-
	7	19259	1062	4,193	0,1	21913	669	4,563	0,1	20348	567	3,845	0,1	19798	4,199
	8	14362	258	3,961	0,1	14669	392	4,182	0,1	19536	578	3,640	0,1	-	-
	9	11215	301	4,197	0,1	10844	345	4,382	0,1	19251	952	3,644	0,1	-	-

References

1. Carmody, W. An easily prepared wide range buffer series. J. Chem. Educ. 40, A386 (1963).
